# Specialist treatment of chronic fatigue syndrome/ME: a cohort study among adult patients in England

**DOI:** 10.1186/s12913-017-2437-3

**Published:** 2017-07-14

**Authors:** Simon M Collin, Esther Crawley

**Affiliations:** School of Social & Community Medicine, Oakfield House, University of Bristol, Oakfield Grove, Bristol, BS8 2BN UK

**Keywords:** Chronic fatigue syndrome, ME, NHS England, Specialist care

## Abstract

**Background:**

NHS specialist chronic fatigue syndrome (CFS/ME) services in England treat approximately 8000 adult patients each year. Variation in therapy programmes and treatment outcomes across services has not been described.

**Methods:**

We described treatments provided by 11 CFS/ME specialist services and we measured changes in patient-reported fatigue (Chalder, Checklist Individual Strength), function (SF-36 physical subscale, Work & Social Adjustment Scale), anxiety and depression (Hospital Anxiety & Depression Scale), pain (visual analogue rating), sleep (Epworth, Jenkins), and overall health (Clinical Global Impression) 1 year after the start of treatment, plus questions about impact of CFS/ME on employment, education/training and domestic tasks/unpaid work. A subset of these outcome measures was collected from former patients 2–5 years after assessment at 7 of the 11 specialist services.

**Results:**

Baseline data at clinical assessment were available for 952 patients, of whom 440 (46.2%) provided 1-year follow-up data. Treatment data were available for 435/440 (98.9%) of these patients, of whom 175 (40.2%) had been discharged at time of follow-up. Therapy programmes varied substantially in mode of delivery (individual or group) and number of sessions. Overall change in health 1 year after first attending specialist services was ‘very much’ or ‘much better’ for 27.5% (115/418) of patients, ‘a little better’ for 36.6% (153/418), ‘no change’ for 15.8% (66/418), ‘a little worse’ for 12.2% (51/418), and ‘worse’ or ‘very much worse’ for 7.9% (33/418). Among former patients who provided 2- to 5-year follow-up (30.4% (385/1265)), these proportions were 30.4% (117/385), 27.5% (106/385), 11.4% (44/385), 13.5% (52/385), and 17.1% (66/385), respectively. 85.4% (327/383) of former patients responded “Yes” to “Do you think that you are still suffering from CFS/ME?” 8.9% (34/383) were “Uncertain”, and 5.7% (22/383) responded “No”.

**Conclusions:**

This multi-centre NHS study has shown that, although one third of patients reported substantial overall improvement in their health, CFS/ME is a long term condition that persists for the majority of adult patients even after receiving specialist treatment.

**Electronic supplementary material:**

The online version of this article (doi:10.1186/s12913-017-2437-3) contains supplementary material, which is available to authorized users.

## Background

Chronic Fatigue Syndrome (CFS), also known as ‘ME’, is a long-term disabling condition characterised by debilitating fatigue of unknown cause, post-exertional malaise, cognitive dysfunction and disturbed/unrefreshing sleep, plus other symptoms including muscle and joint pain, headaches, and dizziness [1]. CFS/ME imposes an immense burden on patients, carers and families [2, 3]. In the UK, adults who attend NHS specialist CFS/ME services have been ill for a median duration of 3 years, and half of those employed at the onset of their illness cease working [4]. A meta-analysis of CFS/ME prevalence studies based on clinically-confirmed cases in several countries gave a prevalence of 0.8% (95% CI 0.2% to 1.3%) [5].

Around 8000 patients are treated annually by NHS specialist CFS/ME services in England [6]. There are approximately 50 such services in England, many of which were established under the CFS/ME Service Investment Programme (2004–2006) [7]. These services follow guidance provided by the National Institute for Health & Care Excellence (NICE), including specific guidelines for diagnosis, specialist care, and ongoing management, with an overall patient-centred approach to treatment [8].

In this study we used initial assessment and patient-reported outcome data from newly referred and former patients who attended specialist CFS/ME services in England to investigate outcomes 1–5 years after initial assessment by the service. We also collected patient-level treatment data to describe variation in treatments across specialist services.

## Methods

### Study cohort - newly referred patients

Newly referred patients were recruited from 11 specialist CFS/ME services across England (10 NHS services, 1 registered independent provider) during the period 01/06/2014 to 30/09/2016. Patients were eligible if they were ≥18 years old and had a CFS/ME diagnosis made or confirmed at an initial clinical assessment appointment in accordance with NICE guidelines [8].

### Patient-level data - newly referred patients

At the time or their initial assessment, patients complete standard questionnaires to obtain quantitative measures of fatigue (Chalder Fatigue Scale [9] and Checklist Individual Strength (CIS20-R) [10]), physical function (RAND SF-36 [11]), general function (Work & Social Adjustment Scale [12]), mood (Hospital Anxiety & Depression Scale (HADS) [13]), pain (visual analogue pain rating scale), daytime sleepiness (Epworth Sleepiness Scale [14]), and sleep problems (Jenkins Sleep Scale [15]). Patients were also asked about the impact of ill health on employment, education/training, and unpaid work (e.g. housework, child care, voluntary work). The same set of questionnaires was sent to patients by post or via email (with a link to online versions of the questionnaires) approximately 12 months after their initial clinical assessment. At follow-up, patients were also asked to rate changes in overall health and CFS/ME (Clinical Global Impression scale), and were asked “Do you think that you are still suffering from CFS/ME?” Patients who didn’t respond were contacted by the clinical team via phone or email on up to 2 further occasions to elicit a response. Data on treatment received by each patient were recorded by clinical teams or extracted from hospital administration databases at the end of the study. Treatment data included the date, duration and type of session (group or individual), and the qualification of the health care professional delivering the session.

### Study cohort - former patients

For 7 of the 11 services, random samples of pseudonymous patient identifiers (50–60 per year for the period 2010–2013) were obtained from the CFS/ME National Outcomes Database (NOD). The CFS/ME NOD is a centralized repository of clinical assessment and patient-reported outcome data which were routinely collected by NHS specialist CFS/ME services across England from 2006 to 2013 for the purpose of service evaluation. Lists of pseudonymous patient identifiers were sent to the clinical teams, who then sent out information sheets, consent forms and questionnaires to patients’ home addresses.

### Patient-level data - former patients

Patients who were treated by specialist services during the period 2010–2013 had already completed questionnaires to provide measures of fatigue (Chalder Fatigue Scale [9]) and physical function (RAND SF-36 [11]) when they were first assessed. At follow-up, former patients were sent these two questionnaires and were asked about changes in overall health and CFS/ME (Clinical Global Impression scale), and whether there had been any changes in employment, education/training, and their ability to do unpaid work/domestic tasks and social/leisure activities. They were also asked “Do you think that you are still suffering from CFS/ME?” Patient-level treatment data for former patients were not extracted from medical records.

### Statistical analysis

Characteristics of patients who provided follow-up data were compared with patients who did not respond using the Chi-squared test for proportions and Kruskal-Wallis test for continuous measures (both α = 0.05). Changes (mean differences) in patient-reported measures between baseline (initial clinical assessment) and 1-year follow-up, and mean patient-reported measures at baseline and 1- to 5-years follow-up were plotted with 95% confidence intervals. Evidence for associations between baseline comorbidities and overall change in health at 2- to 5-year was assessed using the Chi-squared test (α = 0.05).

## Results

### One-year follow-up of newly referred patients

**Fig. 1:**
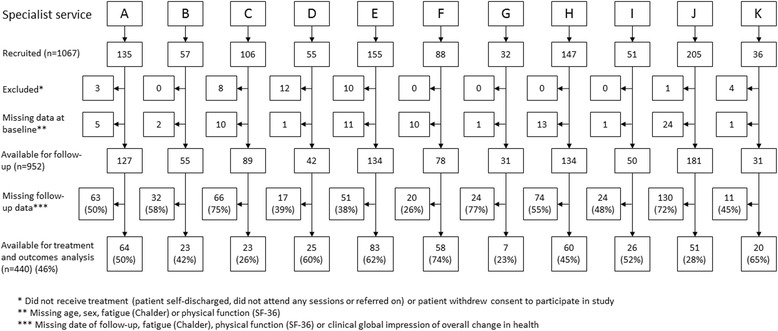
Flowchart showing recruitment, data availability and 1-year follow-up of adult patients across CFS/ME specialist services

Of the 1067 newly referred patients recruited by the 11 services during the study period, 952 (89.2%) had baseline data and were available for follow-up (Fig. 1). Of these patients, 771 (81.0%) were female, the median (IQR) age was 41 (30–50) years, and patients had been ill for 36 (15–84) months. Follow-up data were available for 440/952 (46.2%) of patients, after an interval of 14.0 (12.5–16.7) months. Patients who responded tended to be older (42 (32–51) vs 39 (29–49) years, *p* = 0.003) and to have a shorter self-reported duration of illness (26 (12–80) vs 36 (18–84) months, *p* = 0.02) (Additional file 1: Table S1). Common CFS/ME-related comorbidities tended to occur less frequently among responders vs non-responders: migraine (20.1% (84/417) vs 26.6% (128/482), *p* = 0.02), depression (28.3% (115/406) vs 41.0% (194/473), *p* < 0.001), and anxiety (32.4% (132/407) vs 48.1% (229/476), *p* < 0.001). Conversely, at their initial assessment patients with follow-up were more likely to have been on sick leave (19.0% (74/389) vs 12.8% (56/438), *p* = 0.01) and had shorter working hours per week (21 (8–35) vs 25 (10–37) hours, *p* = 0.06). There were no differences between responders and non-responders in any other characteristics or measures.

Treatment data were available for 77.9% (742/952) of patients with baseline data, and for 98.9% (435/440) of patients with baseline and follow-up data (Table 1). Of patients who provided follow-up data, 40.2% (175/435) did so after they had been discharged. The remaining 59.8% (260/435) had not yet completed treatment or had an open appointment or future follow-up appointment. Four services (D, E, F, and K) achieved ≥60% follow-up. Follow-up at the other services ranged from 23% to 52%. One service (G) had insufficient data (*n* = 7) for analysis of outcomes.

**Table 1:** Treatments received by newly-referred adult CFS/ME patients across CFS/ME specialist services

Service		Median (IQR) total number of sessions	Median (IQR) total session time (hours)	Median (IQR) total individual face-to-face session time (hours)^a^	Median (IQR) total group session time (hours)^a^
A	Treatment data available, *N* = 126	4 (2–8), mean 5.6	6 (3–13), mean 8.2	2 (1–4), mean 3.1	4 (4–12), mean 8.0
	With follow-up data (64/126 (50.8%))	5 (2–8), mean 5.4	6 (4–13), mean 7.9	2 (1–4), mean 3.1	4 (4–12), mean 7.8
	Discharged before follow-up (41/64 (64.1%))	3 (1–5), mean 3.7	5 (1–9), mean 5.8	1 (1–2), mean 2.0	4 (4–12), mean 7.2
B	Treatment data available, *N* = 55	6 (2–10), mean 7.1	6 (2–13), mean 9.5	6 (4–7), mean 6.2	18 (7–24), mean 16.8
	With follow-up data (23/55 (41.8%))	7 (3–12), mean 8.2	7 (3–15), mean 10.7	7 (5–8), mean 7.2	18 (18–28), mean 19.7
	Discharged before follow-up (5/23 (21.7%))	5 (4–5), mean 5.0	5 (4–5), mean 5.0	5 (4–5), mean 5.0	-
C	Treatment data available, *N* = 22^b^	11 (9–12), mean 10.0	19 (17–21), mean 18.4	1 (1–2), mean 1.7	18 (16–20), mean 16.8
	Discharged before follow-up (7/22 (31.8%))	9 (7–11), mean 9.0	17 (13–21), mean 17.0	1 (1–1), mean 1.8	16 (10–20), mean 15.1
D	Treatment data available, *N* = 42	5 (2–8), mean 5.1	4 (3–6), mean 4.7	3 (2–6), mean 4.2	-
	With follow-up data (25/42 (59.5%))	7 (4–8), mean 6.3	6 (4–7), mean 5.7	5 (3–7), mean 5.1	-
	Discharged before follow-up (13/25 (52.0%))	7 (3–8), mean 6.2	5 (4–7), mean 5.6	4 (3–6), mean 4.8	-
E	Treatment data available, *N* = 133	6 (4–7), mean 5.6	6 (4–7), mean 5.9	6 (3–7), mean 5.4	11 (6–12), mean 9.7
	With follow-up data (83/133 (62.4%))	6 (4–7), mean 5.9	6 (4–7), mean 6.1	6 (4–7), mean 5.8	11 (11–11), mean 11.1
	Discharged before follow-up (26/83 (31.3%))	7 (4–7), mean 5.8	7 (4–8), mean 6.2	6 (3–7), mean 5.5	11 (11–14), mean 11.5
F	Treatment data available, *N* = 78	13 (9–14), mean 11.7	15 (10–18), mean 13.9	6 (5–11), mean 7.4	6 (1–13), mean 6.6
	With follow-up data (58/78 (74.4%))	13 (11–15), mean 12.7	17 (13–18), mean 15.2	7 (5–12), mean 8.3	8 (1–13), mean 7.2
	Discharged before follow-up (27/58 (46.6%))	12 (6–13), mean 10.3	16 (7–18), mean 12.9	5 (5–9), mean 5.8	10 (1–13), mean 7.7
G	Treatment data available, *N* = 25	12 (8–21), mean 13.0	12 (7–22), mean 13.8	12 (7–17), mean 13.2	8 (1–15), mean 7.5
	With follow-up data (7/25 (28.0%))	11 (8–25), mean 14.4	10 (8–31), mean 16.3	10 (8–17), mean 14.2	15 (*n* = 1)
	Discharged before follow-up (4/7 (57.1%))	11 (8–18), mean 12.8	10 (7–21), mean 14.0	10 (7–13), mean 10.4	15 (*n* = 1)
H	Treatment data available, *N* = 133	5 (1–9), mean 5.7	No data	No data	No data
	With follow-up data (60/133 (45.1%))	6 (2–11), mean 6.8	No data	No data	No data
	Discharged before follow-up (19/60 (31.7%))	6 (5–9), mean 7.0	No data	No data	No data
I	Treatment data available, *N* = 50	7 (2–10), mean 6.3	9 (3–15), mean 9.1	1 (0.3–6), mean 3.0	14 (8–16), mean 12.2
	With follow-up data (26/50 (52.0%))	9 (5–11), mean 7.9	13 (7–16), mean 11.8	0.3 (0.3–4), mean 2.6	14 (10–17), mean 13.6
	Discharged before follow-up (4/26 (15.4%))	5 (1–7), mean 4.0	5 (1–7), mean 4.0	7 (2–7), mean 5.3	-
J	Treatment data available, *N* = 47^#^	5 (3–11), mean 6.4	4 (3–14), mean 8.7	2 (2–3), mean 2.8	2 (2–2), mean 7.0
	Discharged before follow-up (23/47 (31.8%))	3 (2–4), mean 3.2	3 (2–4), mean 2.9	2 (1–3), mean 1.9	2 (2–2), mean 1.4
K	Treatment data available, *N* = 31	12 (10–13), mean 11.8	20 (16–23), mean 18.2	3 (2–5), mean 3.8	18 (14–20), mean 17.0
	With follow-up data (20/31 (64.5%))	12 (10–15), mean 12.9	20 (17–24), mean 19.1	3 (2–6), mean 3.7	17 (16–20), mean 17.1
	Discharged before follow-up (9/20 (45.0%))	12 (9–13), mean 10.4	18 (17–23), mean 17.7	2 (2–3), mean 2.5	16 (15–21), mean 17.2

^a^ Excluding patients who did not have any sessions of this type

^b^Treatment data were only extracted for patients who had 1-year follow-up data

**Fig. 2:**
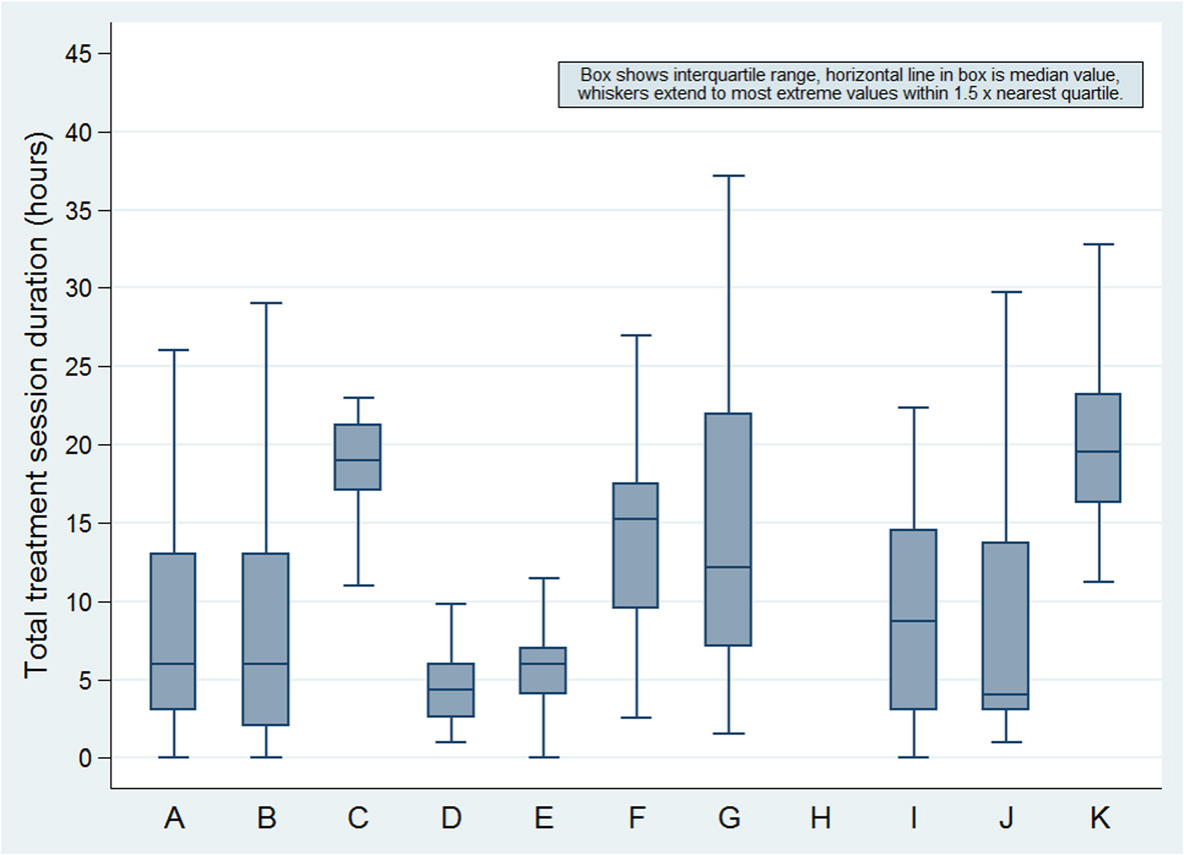
Box chart showing total duration of all treatment sessions (individual face-to-face, individual telephone/internet or group) across CFS/ME specialist services

At 1 year after initial assessment, there was substantial variation in total duration of treatment sessions across services (Fig. 2, Table 1) and in the mix of individual and group sessions, type of health care professional delivery the session and/or content of the session (Additional file 1: Table S2). Median total duration of treatment sessions ranged from 19 to 20 h for services C and K, to 4 h for services D and J. One service (D) provided only individual therapy sessions, two services (E and G) provided mainly (>90%) individual sessions, two services (C and I) mainly group therapy, and the remainder had ratios of individual: group therapy between 50:50 and 60:40. The median duration of therapy sessions was longer among patients with vs without follow-up data (8 (IQR 5–17) vs 6 (3–12), *p* < 0.001) and was shorter among patients who had been discharged vs patients who were still under treatment at the time of follow-up (6 (3–13) vs 9 (6–19), *p* < 0.001).

**Fig. 3:**
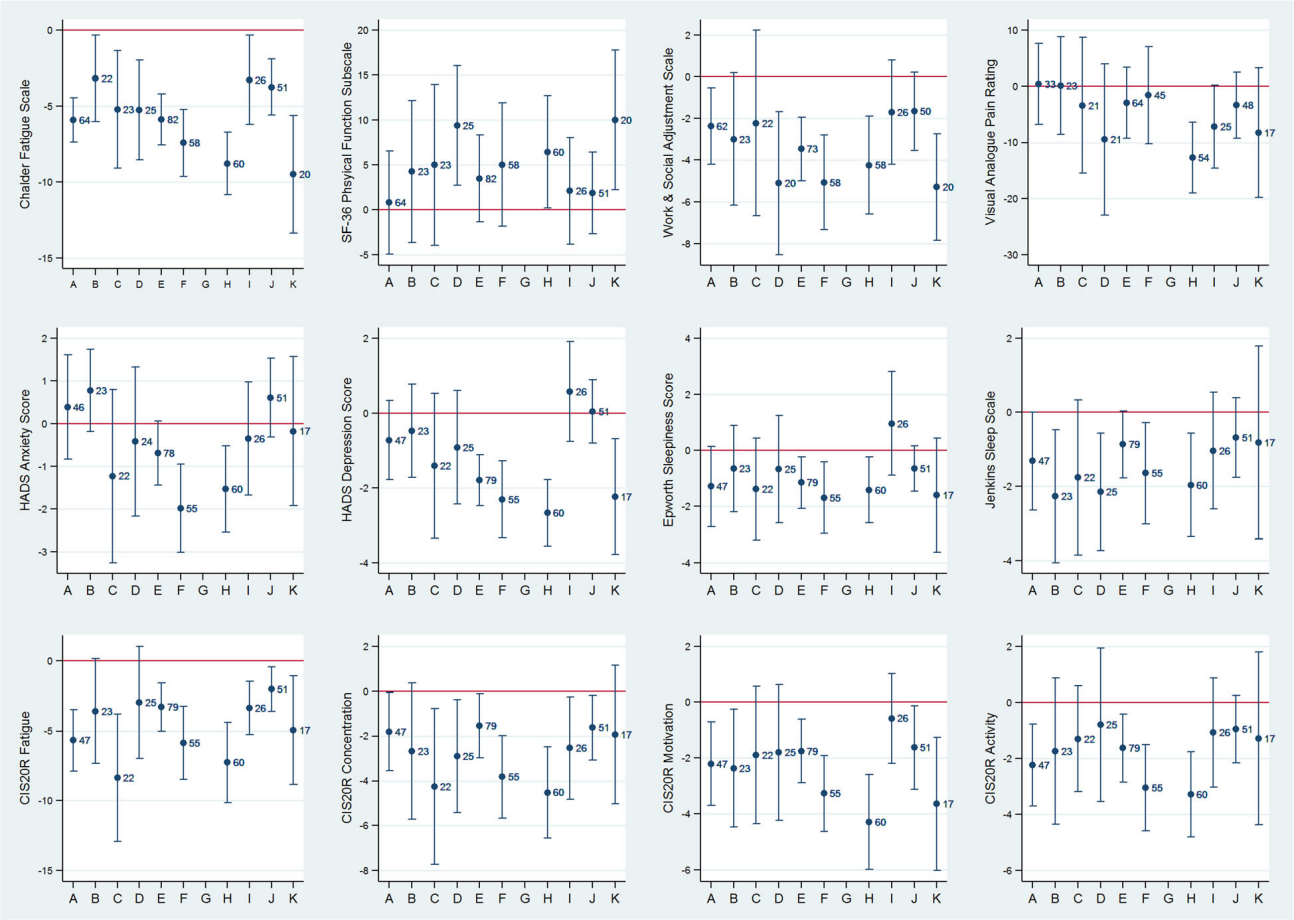
Changes (mean difference with 95% CI) in patient reported outcome measures between initial assessment and 1-year follow-up across CFS/ME specialist services

Changes (mean difference) in patient-reported measures between baseline and follow-up are shown in Fig. 3 and Table 2. There was evidence (*p* < 0.05) of reduction in fatigue on the Chalder Fatigue Scale across all services, with some variation in size of effect. Changes in other outcome measures tended to indicate beneficial effects of treatment, particularly in general function (Work and Social Adjustment Scale), depression, sleep (daytime sleepiness and sleep problems) and concentration and motivation (CIS20R). Evidence for improvements in physical function (SF36), pain, anxiety, and activity (CIS20R) was weaker and less consistent across the services.

**Table 2:** Mean change (95% CI) in patient-reported measures between assessment and 1-year follow-up across CFS/ME specialist services

	A (*n* = 64)	B (*n* = 22)	C (*n* = 23)	D (*n* = 25)	E (*n* = 83)	F (*n* = 58)	H (*n* = 60)	I (*n* = 26)	J (*n* = 51)	K (*n* = 20)
Chalder Fatigue Scale (range 0–33)	−5.92 (−7.38, −4.46)	−3.18 (−6.02, −0.34)	−5.22 (−9.07, −1.36)	−5.24 (−8.53, −1.95)	−5.89 (−7.57, −4.21)	−7.41 (−9.62, −5.21)	−8.78 (−10.8, −6.73)	−3.27 (−6.21, −0.32)	−3.75 (−5.59, −1.90)	−9.50 (−13.7, −5.63)
SF36 Physical Function Subscale (range 0–100)	0.80 (−4.94, 6.53)	4.26 (−3.63, 12.1)	5.00 (−3.93, 13.9)	9.40 (2.72, 16.1)	3.48 (−1.37, 8.32)	5.02 (−1.86, 11.9)	6.45 (0.18, 12.7)	2.12 (−3.82, 8.05)	1.86 (−2.69, 6.41)	10.0 (2.21, 17.8)
Work & Social Adjustment Scale (range 0–40)	−2.37 (−4.20, −0.54)	−3.00 (−6.17, 0.17)	−2.23 (−6.68, 2.23)	−5.10 (−8.53, −1.67)	−3.47 (−4.99, −1.94)	−5.07 (−7.35, −2.79)	−4.24 (−6.58, −1.90)	−1.69 (−4.19, 0.81)	−1.66 (−3.53, 0.21)	−5.30 (−7.85, 2.75)
Visual analogue pain rating scale (range 0–100)	0.42 (−6.82, 7.67)	0.13 (−8.54, 8.80)	−3.38 (−15.5, 8.71)	−9.48 (−23.0, 4.00)	−2.95 (−9.31, 3.40)	−1.60 (−10.2, 7.01)	−12.7 (−19.0, −6.36)	−7.16 (−14.5, 0.23)	−3.38 (−9.27, 2.52)	−8.29 (−19.8, 3.24)
HADS Anxiety Score (range 0–21)	0.39 (−0.83, 1.61)	0.78 (−0.18, 1.74)	−1.23 (−3.25, 0.80)	−0.42 (−2.16, 1.33)	−0.69 (−1.44, 0.06)	−1.98 (−3.01, −0.95)	−1.53 (−2.54, −0.52)	−0.35 (−1.67, 0.98)	0.61 (−0.32, 1.53)	−0.18 (−1.92, 1.56)
HADS Depression Score (range 0–21)	−0.72 (−1.78, 0.34)	−0.48 (−1.73, 0.77)	−1.41 (−3.35, 0.53)	−0.92 (−2.44, 0.60)	−1.80 (−2.48, −1.12)	−2.31 (−3.34, −1.28)	−2.67 (−3.55, −1.78)	0.58 (−0.76, 1.91)	0.04 (−0.81, 0.88)	−2.24 (−3.78, −0.69)
Epworth Sleepiness Scale (range 0–24)	−1.28 (−2.72, 0.15)	−0.65 (−2.19, 0.89)	−1.38 (−3.20, 0.44)	−0.67 (−2.58, 1.25)	−1.15 (−2.07, −0.22)	−1.69 (−2.96, −0.41)	−1.41 (−2.59, −0.23)	0.96 (−0.88, 2.80)	−0.65 (−1.46, 0.16)	−1.59 (−3.63, 0.45)
Jenkins Sleep Jenkins (range 0–20)	−1.32 (−2.64, −0.00)	−2.26 (−4.06, −0.47)	−1.76 (−3.85, 0.33)	−2.15 (−3.74, −0.56)	−0.86 (−1.77, 0.04)	−1.64 (−3.01, −0.27)	−1.97 (−3.36, −0.57)	−1.04 (−2.62, 0.54)	−0.69 (−1.77, 0.39)	−0.82 (−3.43, 1.78)
CIS20R Fatigue Subscale (range 8–56)	−5.67 (−7.89, −3.55)	−3.58 (−7.34, 0.18)	−8.35 (−12.9, −3.78)	−2.95 (−6.97, 1.07)	−3.29 (−5.00, −1.58)	−5.85 (−8.49, −3.22)	−7.25 (−10.1, −4.38)	−3.35 (−5.25, −1.44)	−2.02 (−3.61, −0.43)	−4.94 (−8.85, −1.03)
CIS20R Concentration Subscale (range 5–35)	−1.81 (−3.55, −0.07)	−2.68 (−5.72, 0.35)	−4.25 (−7.73, −0.77)	−2.90 (−5.43, −0.37)	−1.54 (−2.98, −0.11)	−3.82 (−5.66, −1.98)	−4.52 (−6.55, −2.48)	−2.54 (−4.82, −0.25)	−1.63 (−3.07, −0.18)	−1.94 (−5.03, 1.15)
CIS20R Motivation Subscale (range 4–28)	−2.21 (−3.71, −0.72)	−2.37 (−4.48, −0.26)	−1.90 (−4.36, 0.56)	−1.80 (−4.23, 0.63)	−1.75 (−2.90, −0.60)	−3.27 (−4.64, −1.91)	−4.29 (−5.98, −2.59)	−0.58 (−2.18, 1.03)	−1.63 (−3.12, −0.13)	−3.65 (−6.03, −1.26)
CIS20R Activity Subscale (range 3–21)	−2.23 (−3.70, −0.77)	−1.74 (−4.35, 0.87)	−1.30 (−3.19, 0.59)	−0.80 (−3.53, 1.93)	−1.63 (−2.85, −0.42)	−3.05 (−4.60, −1.51)	−3.29 (−4.81, −1.77)	−1.08 (−3.02, 0.87)	−0.96 (−2.17, 0.25)	−1.29 (−4.37, 1.79)

Overall, 18% (71/394) of patients reported returning to work or increasing working hours since first attending a specialist service, whilst 30% (118/394) reported having ceased working or reduced hours because of CFS/ME and 47% (186/394) reported no change (Table 3). Increased ability to perform unpaid work and domestic tasks was reported by 35% (129/372) of patients, reduced ability by 34% (127/372), and no change by 29% (109/372). Similarly, 35% (139/397) of patients reported being able to do more social and leisure activities, 36% (143/397) reported being less able, and 26% (102/397) reported no change. Only 5% (11/240) reported returning to, or increasing hours of education/training, whilst 13% (31/240) ceased or reduced their hours and 79% (189/240) reported no change.

**Table 3:** Change in paid and unpaid activities across CFS/ME specialist services

		A	B	C	D	E	F	H	I	J	K	Overall
Paid work (employed/self-employed) since attending CFS/ME service	There has been no change in my employment situation	58.7% (27/46)	34.8% (8/23)	42.9% (9/21)	37.5% (9/24)	40.0% (30/75)	45.1% (23/51)	37.0% (20/54)	53.9% (14/26)	60.8% (31/51)	70.6% (12/17)	47.2% (186/394)
	I have been able to return to work or increase my hours	13.0% (6/46)	26.1% (6/23)	19.1% (4/21)	16.7% (4/24)	14.7% (11/75)	19.6% (10/51)	29.6% (16/54)	15.4% (4/26)	11.8% (6/51)	11.8% (2/17)	18.0% (71/394)
	I have stopped working or reduced my hours because of CFS/ME	23.9% (11/46)	39.1% (9/23)	23.8% (5/21)	33.3% (8/24)	36.0% (27/75)	31.4% (16/51)	31.5% (17/54)	26.9% (7/26)	27.5% (14/51)	17.7% (3/17)	30.0% (118/394)
	I have stopped working or reduced my hours for other reasons	4.4% (2/46)	0.0% (0/23)	14.3% (3/21)	12.5% (3/24)	9.3% (7/75)	3.9% (2/51)	1.9% (1/54)	3.9% (1/26)	0.0% (0/51)	0.0% (0/17)	4.8% (19/394)
Education or training since attending CFS/ME service	There has been no change in my college/university attendance	90.3% (28/31)	100.0% (9/9)	73.7% (14/19)	66.7% (10/15)	70.7% (29/41)	73.0% (27/37)	74.1% (20/27)	85.7% (12/14)	93.6% (29/31)	80.0% (8/10)	78.8% (189/240)
	I have been able to return to education/training or increase my hours	6.5% (2/31)	0.0% (0/9)	5.3% (1/19)	6.7% (1/15)	0.0% (0/41)	10.8% (4/37)	3.7% (1/27)	0.0% (0/14)	0.0% (0/31)	0.0% (0/10)	4.6% (11/240)
	I have stopped attending or reduced my hours because of CFS/ME	3.2% (1/31)	0.0% (0/9)	10.5% (2/19)	20.0% (3/15)	24.4% (10/41)	13.5% (5/37)	14.8% (4/27)	14.3% (2/14)	3.2% (1/31)	20.0% (2/10)	12.9% (31/240)
	I have stopped attending or reduced my hours for other reasons	0.0% (0/31)	0.0% (0/9)	10.5% (2/19)	6.7% (1/15)	4.9% (2/41)	2.7% (1/37)	7.4% (2/27)	0.0% (0/14)	3.2% (1/31)	0.0% (0/10)	3.8% (9/240)
Unpaid work and domestic tasks (childcare, housework, voluntary work, driving, cooking, cleaning, etc.) since attending CFS/ME service	My ability to do unpaid work and domestic tasks has not changed	25.6% (11/43)	30.0% (6/20)	36.4% (8/22)	30.4% (7/23)	27.7% (18/65)	30.6% (15/49)	34.6% (18/52)	30.8% (8/26)	28.0% (14/50)	25.0% (4/16)	29.3% (109/372)
	I have been able to do more unpaid work and domestic tasks	48.8% (21/43)	30.0% (6/20)	27.3% (6/22)	26.1% (6/23)	24.6% (16/65)	38.8% (19/49)	38.5% (20/52)	26.9% (7/26)	34.0% (17/50)	43.8% (7/16)	34.7% (129/372)
	I do less unpaid work and/or fewer tasks because of CFS/ME	23.3% (10/43)	40.0% (8/20)	31.8% (7/22)	44.6% (29/23)	44.6% (29/65)	26.5% (13/49)	26.9% (14/52)	42.3% (11/26)	38.0% (38/50)	25.0% (4/16)	34.1% (127/372)
	I do less unpaid work and/or fewer tasks for other reasons	2.3% (1/43)	0.0% (0/20)	4.6% (1/22)	0.0% (0/23)	3.1% (2/65)	4.1% (2/49)	0.0% (0/52)	0.0% (0/26)	0.0% (0/50)	6.3% (1/16)	1.9% (7/372)
Social and leisure activities (going out, inviting people over, hobbies, gardening, travel, exercise, etc.) since attending CFS/ME service	My ability to do social & leisure activities has not changed	21.3% (10/47)	21.7% (5/23)	27.3% (6/22)	40.0% (8/20)	21.9% (16/73)	35.2% (19/54)	25.4% (15/59)	23.1% (6/26)	25.5% (13/51)	18.8% (3/16)	25.7% (102/397)
	I have been able to do more social & leisure activities	46.8% (22/47)	43.5% (10/23)	27.3% (6/22)	20.0% (4/20)	28.8% (21/73)	35.2% (19/54)	40.7% (24/59)	30.8% (8/26)	29.4% (15/51)	43.8% (7/16)	35.0% (139/397)
	I do fewer social & leisure activities because of CFS/ME	29.8% (14/47)	34.8% (8/23)	27.3% (6/22)	40.0% (8/20)	45.2% (33/73)	27.8% (15/54)	28.8% (17/59)	46.2% (12/26)	45.1% (23/51)	31.3% (5/16)	36.0% (143/397)
	I do fewer social & leisure activities for other reasons	2.1% (1/47)	0.0% (0/23)	18.2% (4/22)	0.0% (0/20)	4.1% (3/73)	1.9% (1/54)	5.1% (3/59)	0.0% (0/26)	0.0% (0/51)	6.3% (1/16)	3.3% (13/397)

**Fig. 4:**
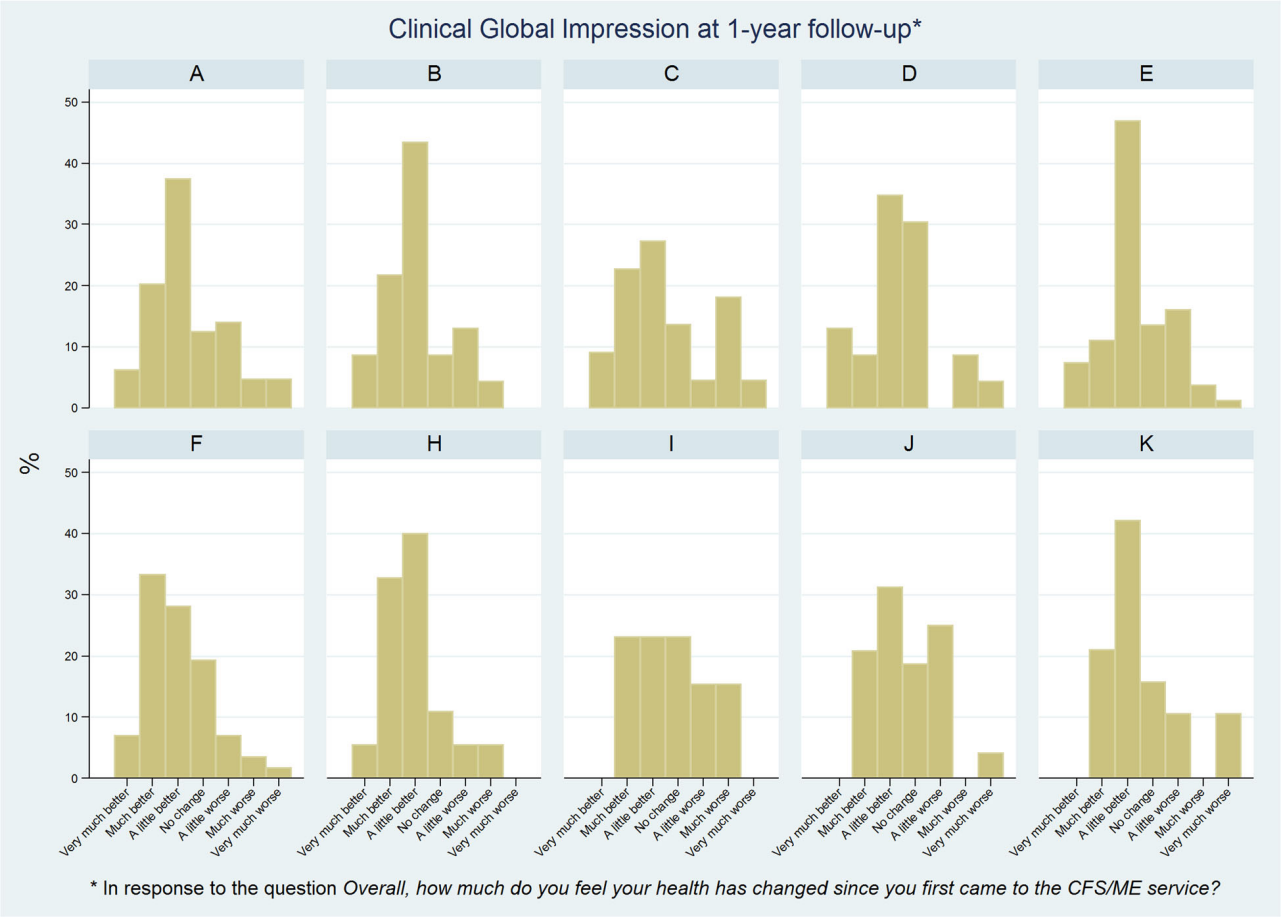
Clinical Global Impression responses at 1-year follow-up across CFS/ME specialist services

Overall changes in health were reasonably consistent across the specialist services, with 28% (115/418) reporting their health as much better or very much better, 65% (270/418) reporting little or no change, and 8% (33/418) reporting much worse or very much worse health (Table 4, Fig. 4). Of those reporting little or no change, the majority said that they were ‘a little better’ (36.6% (153/418)), with 15.8% (66/418) indicating ‘no change’ and 12.2% (51/418) a slight deterioration. Similar proportions were observed in response to the question “Overall, how much do you feel your CFS/ME has changed since you first came to the service?” In response to the question “Do you think that you are still suffering from CFS/ME?” 87% (341/391) responded “Yes”, 3% (11/391) “No” and 10% (39/391) were “Uncertain”. Of those who responded “Yes”, 23% (78/339) also said that their overall health was much or very much better, compared with 82% (9/11) of those who responded “No” and 58% (21/36) of those who were “Uncertain”. Changes (mean difference) in patient-reported measures between baseline and follow-up for each level of overall improvement in health are provided as supplementary information (Additional file 2: Figure S1 and Additional file 1: Table S3).

**Table 4:** Overall change in health and CFS/ME across CFS/ME specialist services

		A	B	C	D	E	F	H	I	J	K	Overall
Overall, how much do you feel your health has changed since you first came to the CFS/ME service?	Much better or very much better	26.6% (17/64)	30.4% (7/23)	31.8% (7/22)	21.7% (5/23)	18.5% (15/81)	40.4% (23/57)	38.2% (21/55)	23.1% (6/26)	20.8% (10/48)	21.1% (4/19)	27.5% (115/418)
	A little better, no change or a little worse	64.1% (41/64)	65.2% (15/23)	45.5% (10/22)	65.2% (15/23)	76.5% (62/81)	54.4% (31/57)	56.4% (31/55)	61.5% (16/26)	75.0% (36/48)	68.4% (13/19)	64.6% (270/418)
	Much worse or very much worse	9.4% (6/64)	4.4% (1/23)	22.7% (5/22)	13.0% (3/23)	4.9% (4/81)	5.3% (3/57)	5.5% (3/55)	15.4% (4/26)	4.2% (2/48)	10.5% (2/19)	7.9% (33/418)
Overall, how much do you feel your CFS/ME has changed since you first came to the CFS/ME service?	Much better or very much better	23.8% (15/63)	30.4% (7/23)	31.8% (7/22)	20.0% (4/20)	21.2% (14/66)	35.1% (20/57)	34.6% (19/55)	23.1% (6/26)	12.8% (6/47)	33.3% (6/18)	26.2% (104/397)
	A little better, no change or a little worse	71.4% (45/63)	60.9% (14/23)	59.1% (13/22)	65.0% (13/20)	72.7% (48/66)	56.1% (32/57)	61.8% (34/55)	69.2% (18/26)	76.6% (36/47)	61.1% (11/18)	66.5% (264/397)
	Much worse or very much worse	4.8% (3/63)	8.7% (2/23)	9.1% (2/22)	15.0% (3/20)	6.1% (4/66)	8.8% (5/57)	3.6% (2/55)	7.7% (2/26)	10.6% (5/47)	5.6% (1/18)	7.3% (29/397)
Do you think that you are still suffering from CFS/ME?	Yes	78.7% (37/47)	81.8% (18/22)	81.0% (17/21)	85.0% (17/20)	90.4% (66/73)	87.3% (48/55)	86.4% (51/59)	92.3% (24/26)	92.2% (47/51)	94.1% (16/17)	87.2% (341/391)
	No	4.3% (2/47)	4.6% (1/22)	0.0% (0/21)	10.0% (2/20)	2.7% (2/73)	1.8% (1/55)	0.0% (0/59)	3.9% (1/26)	3.9% (2/51)	0.0% (0/17)	2.8% (11/391)
	Uncertain	17.0% (8/47)	13.6% (3/22)	19.1% (4/21)	5.0% (1/20)	6.9% (5/73)	10.9% (6/55)	13.6% (8/59)	3.9% (1/26)	3.9% (2/51)	5.9% (1/17)	10.0% (39/391)

### Long-term (2- to 5-year) follow-up of former patients

**Fig. 5:**
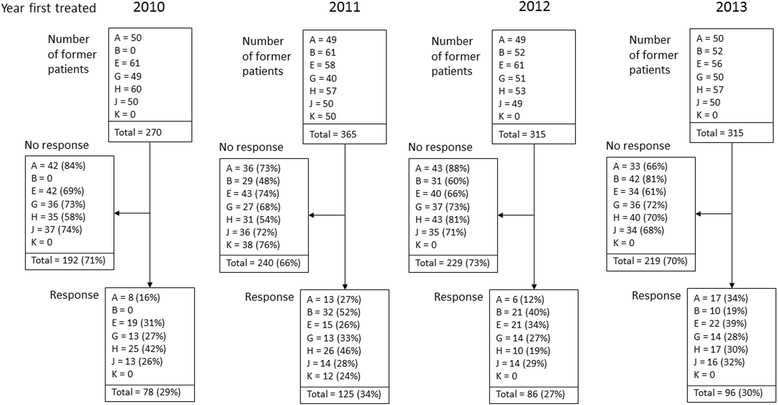
Flowchart showing follow-up of former patients treated 2–5 years previously by CFS/ME specialist services

Seven of the specialist services attempted to contact random samples of former patients who were assessed and treated in 2010, 2011, 2012 or 2013 (total *N* = 1265), and long-term follow-up questionnaires were returned by 30% (385/1265) of these patients (Fig. 5). Baseline characteristics (at time of assessment) were the same in patients who did vs did not respond, except that patients who responded tended to be older (43 (33–51) vs 38 (30–47) years, *p* < 0.001) (Additional file 1: Table S4), and less likely to have presented with comorbid depression (26.6% (89/335) vs 34.8% (247/709), *p* = 0.008). Otherwise, there were no differences between responders and non-responders in any other baseline characteristics or measures. There was no difference between the two groups in their responses to the Clinical Global Impression question “Overall, how much do you feel your health has changed since you first came to the CFS/ME service?” at 1 year follow-up, with 32% (84/263) of non-responders vs 38% (84/223) of responders indicating that they were very much or much better, 58% (153/263) vs 56% (124/223) reporting little or no change, and 10% (26/263) vs 7% (15/223) indicating that they were very much or much worse (*p* = 0.26).

**Fig. 6:**
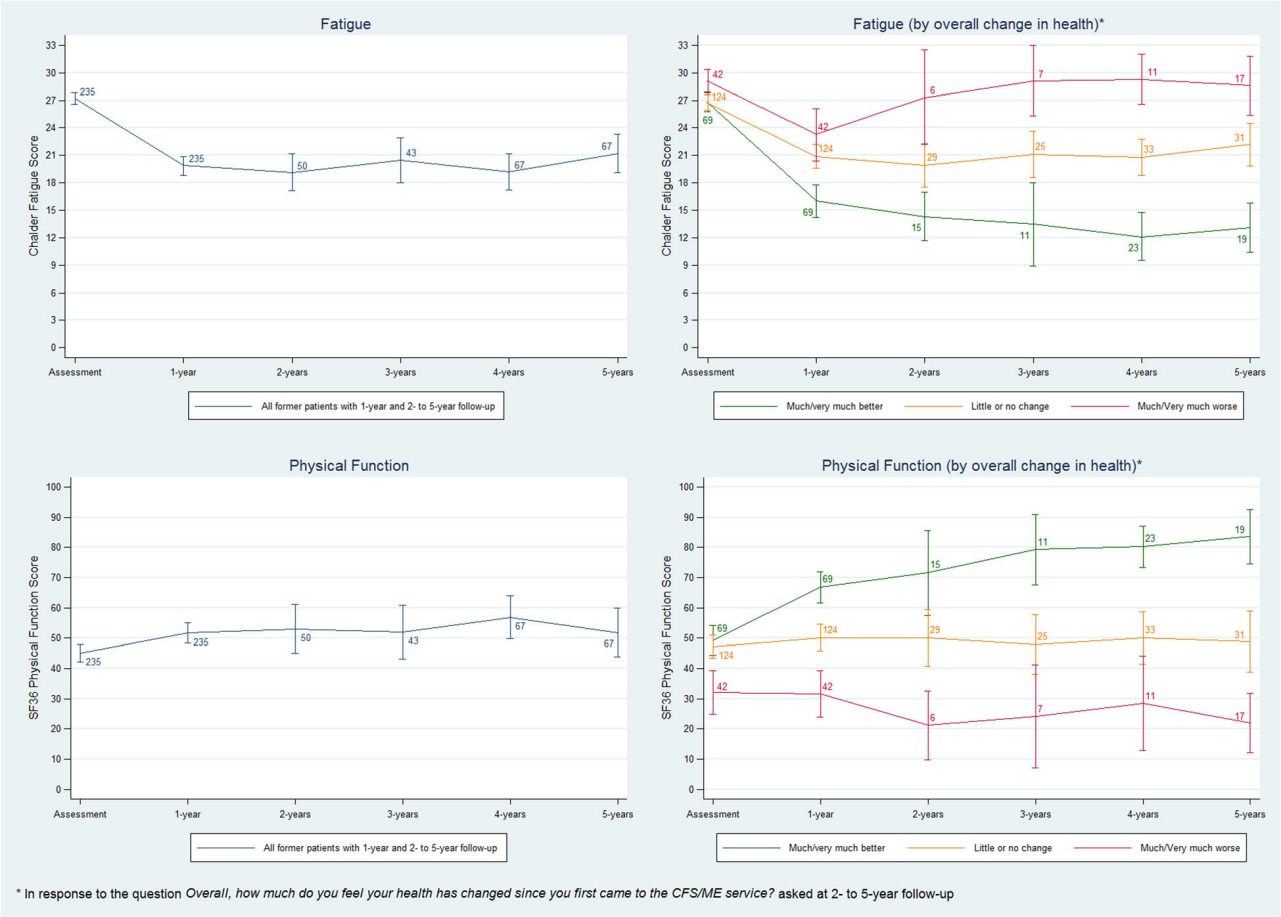
Trends in patient-reported fatigue (Chalder) and physical function (SF36) from initial assessment up to 5 years follow-up among patients treated by CFS/ME specialist services

Patient-reported measures of fatigue and physical function showed no overall change from 1 year follow-up onwards (Table 5, Fig. 6), but stratification by responses to the Clinical Global Impression questionnaire at 2 to 5 years post-assessment showed that 30–40% of patients improved from 1 year onwards, 40–60% experienced little or no change, and 20–25% deteriorated (Fig. 6). Over the 2–5 year follow-up period, 24% (88/372) of former patients reported being able to return to work or increase their working hours, 12% (29/238) returned to, or increased their hours of, education, 36% (132/365) increased unpaid work and domestic tasks, and 35% (133/382) increased their social and leisure activities (Table 6). Conversely, 27% (102/372), 13% (29/238), 32% (117/365), and 39% (149/382) reported stopping or reducing paid work, education/training, unpaid/domestic work, and social/leisure activities, respectively.

**Table 5:** Median (IQR) fatigue and physical function at baseline (assessment) and at 1- to 5-year follow-up among patients treated by CFS/ME specialist services

		Baseline	1 year	2 years	3 years	4 years	5 years
Chalder Fatigue Scale (range 0–33)	All former patients in sample	28 (24–31), *n* = 1265	21 (13–26), *n* = 516	20 (15–27), *n* = 80	21 (15–26), *n* = 83	20 (16–26), *n* = 106	21 (14–28), *n* = 99
	Patients who reported very much or much better overall health at 2, 3, 4, or 5 years	27 (23–40), *n* = 117	16 (11–22), *n* = 69	15.5 (11–20), *n* = 20	14 (8–19), *n* = 22	16 (11–19), *n* = 41	11 (11–17), *n* = 31
	Patients who reported little or no change in overall health at 2, 3, 4, or 5 years	27.5 (23–30), *n* = 202	22 (16–26), *n* = 124	20.5 (15.5–27), *n* = 52	22 (18–26), *n* = 47	21 (17–24), *n* = 47	21 (19–27), *n* = 43
	Patients who reported very much or much worse overall health at 2, 3, 4, or 5 years	27 (33–66), *n* = 30	24.5 (19–32), *n* = 42	30 (24–32), *n* = 8	33 (26–33), *n* = 14	30.5 (26–33), *n* = 18	31 (25–33), *n* = 25
SF36 Physical Function Subscale (range 0–100)	All former patients in sample	47 (27–67), *n* = 1265	52 (32–77), *n* = 516	52 (29.5–74.5), *n* = 80	52 (27–77), *n* = 83	57 (32–82), *n* = 106	52 (32–82), *n* = 99
	Patients who reported very much or much better overall health at 2, 3, 4, or 5 years	52 (37–67), *n* = 117	72 (52–82), *n* = 69	77 (49.5–97), *n* = 20	87 (67–97), *n* = 22	82 (62–87), *n* = 41	87 (72–97), *n* = 31
	Patients who reported little or no change in overall health at 2, 3, 4, or 5 years	47 (32–62), *n* = 202	49.5 (32–72), *n* = 124	52 (29.5–72), *n* = 52	47 (27–67), *n* = 47	52 (27–67), *n* = 47	52 (32–72), *n* = 43
	Patients who reported very much or much worse overall health at 2, 3, 4, or 5 years	29.5 (17–52), *n* = 66	24.5 (12–52), *n* = 42	24.5 (7–34.5), *n* = 8	17 (2–37), *n* = 14	22 (7–47), *n* = 18	22 (2–37), *n* = 25

]

**Table 6:** Change in activities and health across CFS/ME specialist services at 2- to 5-year follow-up

		2 years	3 years	4 years	5 years	Overall
Paid work (employed/self-employed) since attending CFS/ME service	There has been no change in my employment situation	51.3% (39/76)	45.0% (36/80)	41.8% (43/103)	26.8% (26/97)	41.4% (154/372)
	I have been able to return to work or increase my hours	18.4% (14/76)	17.5% (14/80)	28.2% (29/103)	30.9% (30/97)	23.7% (88/372)
	I have stopped working or reduced my hours because of CFS/ME	26.3% (20/76)	28.8% (23/80)	22.3% (23/103)	32.0% (31/97)	27.4% (102/372)
	I have stopped working or reduced my hours for other reasons	4.0% (3/76)	8.8% (7/80)	7.8% (8/103)	10.3% (10/97)	7.5% (28/372)
Education or training since attending CFS/ME service	There has been no change in my college/university attendance	57.8% (26/45)	69.0% (40/58)	69.2% (45/65)	74.1% (43/58)	67.7% (161/238)
	I have been able to return to education/training or increase my hours	11.1% (5/45)	10.3% (6/58)	15.4% (10/65)	10.3% (6/58)	12.2% (29/238)
	I have stopped attending or reduced my hours because of CFS/ME	17.8% (8/45)	17.2% (10/58)	7.7% (5/65)	8.6% (5/58)	12.6% (30/238)
	I have stopped attending or reduced my hours for other reasons	13.3% (6/45)	3.5% (2/58)	7.7% (5/65)	6.9% (4/58)	7.6% (18/238)
Unpaid work and domestic tasks (childcare, housework, voluntary work, driving, cooking, cleaning, etc.) since attending CFS/ME service	My ability to do unpaid work and domestic tasks has not changed	26.3% (20/76)	31.3% (25/80)	24.8% (25/101)	30.1% (28/93)	29.3% (107/365)
	I have been able to do more unpaid work and domestic tasks	29.0% (22/76)	33.8% (27/80)	42.6% (43/101)	38.7% (36/93)	36.2% (132/365)
	I do less unpaid work and/or fewer tasks because of CFS/ME	39.5% (30/76)	35.0% (28/80)	30.7% (31/101)	28.0% (26/93)	32.1% (117/365)
	I do less unpaid work and/or fewer tasks for other reasons	5.3% (4/76)	0.0% (0/80)	2.0% (2/101)	3.2% (3/93)	2.5% (9/365)
Social and leisure activities (going out, inviting people over, hobbies, gardening, travel, exercise, etc.) since attending CFS/ME service	My ability to do social & leisure activities has not changed	25.0% (20/80)	34.6% (28/81)	14.2% (15/106)	22.5% (22/98)	23.0% (88/382)
	I have been able to do more social & leisure activities	28.8% (23/80)	32.1% (26/81)	37.7% (40/106)	37.8% (37/98)	34.8% (133/382)
	I do fewer social & leisure activities because of CFS/ME	43.8% (35/80)	32.1% (26/81)	43.4% (46/106)	35.7% (35/98)	39.0% (149/382)
	I do fewer social & leisure activities for other reasons	2.5% (2/80)	1.2% (1/81)	4.7% (5/106)	4.1% (4/98)	3.1% (12/382)
Overall, how much do you feel your health has changed since you first came to the CFS/ME service?	Much better or very much better	25.0% (20/80)	26.5% (22/83)	38.7% (41/106)	31.3% (31/99)	30.4% (117/385)
	A little better, no change or a little worse	65.0% (52/80)	56.6% (47/83)	44.3% (47/106)	43.4% (43/99)	52.5% (202/385)
	Much worse or very much worse	10.0% (8/80)	16.9% (14/83)	17.0% (18/106)	25.3% (25/99)	17.1% (66/385)
Overall, how much do you feel your CFS/ME has changed since you first came to the CFS/ME service?	Much better or very much better	18.8% (15/80)	28.9% (24/83)	41.0% (43/105)	35.1% (34/97)	31.4% (120/382)
	A little better, no change or a little worse	73.8% (59/80)	57.8% (48/83)	41.0% (43/105)	43.3% (42/97)	53.1% (203/382)
	Much worse or very much worse	7.5% (6/80)	13.3% (11/83)	18.1% (19/105)	21.7% (21/97)	15.5% (59/382)
Do you think that you are still suffering from CFS/ME?	Yes	86.3% (69/80)	90.4% (75/83)	82.1% (87/106)	82.7% (81/98)	85.4% (327/383)
	No	5.0% (4/80)	2.4% (2/83)	6.6% (7/106)	8.2% (8/98)	5.7% (22/383)
	Uncertain	8.8% (7/80)	7.2% (6/83)	11.3% (12/106)	9.2% (9/98)	8.9% (34/383)

Approximately half of former patients (52.5% (202/385)) reported little or no overall change in their health (of whom half said that they were a little better), one third (30.4% (117/385)) said that they were very much or much better, and 17% (66/385) rated their health as very much or much worse. The majority of former patients (85% (327/383)) responded affirmatively to the question “Do you think that you are still suffering from CFS/ME?”, 6% (22/383) said “No”, and 9% (34/383) were “Uncertain”. Of those who responded “Yes”, 23% (74/327) also said that their overall health was much or very much better, compared with 95% (21/22) of those who responded “No” and 62% (21/34) of those who were “Uncertain”.

Overall change in health at 2- to 5-year follow-up was associated with comorbid fibromyalgia (*p* = 0.005) and depression (*p* = 0.02) recorded at the patient’s baseline clinical assessment, but was not associated with comorbid anxiety, migraine, or irritable bowel syndrome. Very much or much better health was reported by 15.7% (13/83) vs 34.4% (86/250) of patients who did vs did not have comorbid fibromyalgia, and by 22.5% (20/89) vs 32.1% (79/246) of patients with vs without comorbid depression. Very much or much worse health was reported by 21.7% (18/83) vs 14.8% (37/250) of patients with vs without fibromyalgia, and by 25.8% (23/89) vs 13.4% (33/246) of patients with vs without comorbid depression.

## Discussion

This study has described substantial variation in total therapy time for adult patients attending NHS specialist CFS/ME services in England. Changes in patient-reported outcome measures approximately one year after patients’ initial assessments demonstrated clear improvement, particularly in fatigue, general function, depression, sleep, concentration, and motivation. At one year follow-up, one quarter of patients rated their overall health as very much or much better, the majority (two thirds) reported little or no change (of whom half had improved slightly), and <10% reported much or very much worse health. Just under 90% of patients said that they were still suffering from CFS/ME, although one quarter of these also said that their overall health was very much or much better. Among former patients who returned long term (2- to 5-year) follow-up questionnaires, one third rated their overall health as very much or much better, half reported little or no change, 17% reported substantial deterioration, and 85% said that they were still suffering from CFS/ME.

### Strengths and limitations

The main strength of our study is that a large cohort of patients was recruited from NHS specialist services in England, all of which follow NICE guidelines for the diagnosis and management of CFS/ME [8]. This should ensure that the patients in our study have been diagnosed with CFS/ME according to the same criteria, namely persistent or recurrent debilitating fatigue of ≥4 months’ duration which is not lifelong, or the result of ongoing exertion, or alleviated by rest, or explained by other conditions, and which results in a substantial reduction in activity. We measured patient-reported outcomes using standardised questionnaires which are used routinely in clinical practice, and which have also been used widely in epidemiological research [16, 17].

The main limitation of this study is the substantial loss to follow-up, both among the newly referred patients and among the former patients. Although 12-month follow-up of newly referred patients was 74% at one service and 60–65% at three services, follow-up at the other seven services ranged from 23 to 52%. Long-term follow-up of former patients was 30%. These losses to follow-up mean that all of our analyses must be interpreted with the caveat that bias could be introduced in either direction if patients who were more (or less) likely to recover were more (or less) likely to return their questionnaires. Another limitation is that we measured outcomes in newly-referred patients at a fixed time after their initial assessment, with 60% of patients still being under treatment at time of follow-up. Also, measurement of outcomes at a single time point is not ideal for an illness which can fluctuate over time.

### Comparison with previous literature

The best comparators for our real world results are the results reported by the PACE trial of cognitive behaviour therapy (CBT) and graded exercise therapy (GET) for adults with CFS/ME. The PACE trial showed a reduction (in the CBT and GET arms) in mean fatigue (on the Chalder Scale) from 28 points at randomisation to 20 points at 12 months [18], followed by a slight (1–2 point) further reduction at 31 (IQR 30–32) months follow-up [19]. This is very similar to the trend observed in our data. PACE reported larger improvements in physical function (from 37 to 39 points at baseline to 58 points at 12 months followed by slight or no further change at long-term follow-up) than observed in our study, a difference between trial and real world outcomes which we found previously in an analysis of routinely-collected data [20]. The proportions of PACE trial participants (in the CBT and GET arms) who rated their overall health as very much or much better at 12 and 31 months were 41% and 42–48%, respectively, compared with 28% and 30% in our study (at 12 months and 2–5 years).

We cannot say whether these differences represent better long term outcomes attributable to the treatment programmes in the trial, the effect of the majority of patients in our study still being under treatment, or bias in our study if patients who improved were more likely to be lost to follow up. Participants in the CBT and GET arms of the PACE trial received (median (IQR)) 14 (12–15) individual CBT sessions (86% face-to-face, 14% by telephone) and 13 (12–14) individual GET sessions (94% face-to-face, 6% by telephone) respectively, and one fifth went on to receive further therapy sessions after the trial ended. Only four services in our study (C, F, G, and K) provided a similar number of therapy sessions, with considerable variation in follow-up and outcomes. One of these services (F) provided 12 (6–13) therapy sessions and follow-up was obtained from 74% (58/78) of patients of whom 40% (23/57) rated their overall health as very much or much better. In this service, the ratio of individual: group therapy was 50:50, which suggests scope for even better cost-effectiveness than reported in the PACE trial [21].

### Implications for clinical practice and future research

Pointers for future research are perhaps given by some of the between-service differences in outcomes. For example, patients who were treated by service D (with 60% follow-up) reported particularly good improvements in physical function despite receiving a relatively small number (median 7 (IQR 3–8)) of (mostly GET) sessions - this service also delivered 28% of therapy sessions via telephone. Service K (with 65% follow-up) obtained similar improvements in physical function by means of a larger number of sessions (12 (9–13), mostly with an occupational therapist) of which 65% were group sessions. These similarities in outcomes and differences in modes of delivery merit further exploration, and could form the basis of future randomised controlled trials. Similarly, treatments in our study were delivered by multidisciplinary teams variously comprising specialist clinicians, clinical psychologists, occupational therapists, and physiotherapists, which highlights the lack of an evidence base regarding the optimal mix of health care professionals required to deliver the most cost-effective service. Factorial trials are particularly suited to addressing multiple questions about content and delivery of treatments [22], and these could also be designed (by randomising predefined subgroups of patients with specific comorbidities and/or symptom profiles) to investigate the big question in CFS/ME treatment, namely why some patients experience substantial improvement whilst others report little or no improvement.

Qualitative research has shown that specialist CFS/ME services play an important role in patients’ journeys towards improved quality of life [23, 24]. Anecdotally, services report overall high patient satisfaction, which may appear to be at odds with a minority of patients experiencing substantial improvement. This apparent paradox may be explained in part by the difficulty of measuring long-term outcomes in a complex chronic illness [16, 25], a problem which could perhaps be addressed by using objective rather than subjective measures [26]. We might also consider how outcomes in our study compare with other chronic illnesses, such as chronic widespread pain, fibromyalgia, arthritis and multiple sclerosis [22, 27, 28], and with the poor prognosis for CFS/ME in adults if no specialist treatment is received. Our qualitative study was set in three of the eleven services in the present study [23]. It showed that referral to a CFS/ME specialist service was typically the first positive step in coming to terms with a life changing contested illness, with improvement coming about through a process which included validation of patients’ experiences, acceptance of change, practical advice and support, and therapeutic outcomes. Professional support was an important theme, and we recognize that some of the between-service differences in outcomes in the present study may be attributable to therapist effects [29–31].

## Conclusions

This multi-centre study in the NHS has shown that CFS/ME is a long term condition that persists for the majority of adult patients even after receiving specialist treatment. Whilst 50–65% experienced little or no change in their condition 1–5 years after accessing a specialist service and 10–20% reported a deterioration, up to 30% of patients reported very much or much better health (and the majority of those who experienced little or no change had improved slightly). Given the adverse impact of CFS/ME on patients and their families, substantial improvement in 20–30% of the approximately 8000 patients treated each year by specialist CFS/ME services in England represents a large individual and societal benefit, and supports the argument that services need to be sufficiently resourced to treat promptly all newly diagnosed patients according to individual need.

## Additional files

Additional file 1: Table S1. Characteristics of newly-referred patients with and without 12-month follow-up data across CFS/ME specialist services. **Table S2.** Treatments received by newly-referred patients across CFS/ME specialist services. **Table S3.** Mean change (95% CI) in patient-reported outcome measures between assessment and 1-year follow-up, categorised by overall improvement in health. **Table S4.** Baseline characteristics of former patients with and without 2- to 5-year follow-up data across CFS/ME specialist services. (DOCX 27 kb)

Additional file 2: Figure S1. Changes (mean difference with 95% CI) in patient reported measures between initial assessment and 1-year follow-up by overall improvement in health among patients treated by CFS/ME specialist services. (TIFF 12271 kb)
