# Supplementary material for: Specialist treatment of chronic fatigue syndrome/ME: a cohort study among adult patients in England
Source: BMC Health Serv Res. 2017 Jul 14;17:488. doi: 10.1186/s12913-017-2437-3 (PMC5513420; doi:10.1186/s12913-017-2437-3)
Supplement: Supplementary file 1 — Characteristics of newly-referred patients with and without 12-month follow-up data across CFS/ME specialist services. Table S2. Treatments received by newly-referred patients across CFS/ME specialist services. Table S3. Mean change (95% CI) in patient-reported outcome measures between assessment and 1-year follow-up, categorised by overall improvement in health. Table S4. Baseline characteristics of former patients with and without 2- to 5-year follow-up data across CFS/ME specialist services. (DOCX 27 kb) [file 12913_2017_2437_MOESM1_ESM.docx]

Table S1: Characteristics of newly-referred patients with and without 12-month follow-up data across CFS/ME specialist services

| Service | 1-year follow-up | Number of patients | Age (years) | Female | Chalder Fatigue score (range 0-33) | SF-36 physical function score (range 0-100) | Duration of illness (months) |
| --- | --- | --- | --- | --- | --- | --- | --- |
|  |  | n (%) | median (IQR) | n (%) | median (IQR) | median (IQR) | median (IQR) |
| A | No | 63 (49.6%) | 36 (25-47) | 56 (88.9%) | 28 (24-31) | 35 (20-65) | 36 (24-96), n=37 |
|  | Yes | 64 (50.4%) | 42 (32-49)* | 52 (81.3%) | 28 (23-30) | 50 (23-65) | 24 (12-60), n=34* |
| B | No | 32 (58.2%) | 39 (29-49) | 23 (71.9%) | 28 (23-31) | 58 (28-69) | 28 (12-72), n=30 |
|  | Yes | 23 (41.8%) | 40 (32-48) | 20 (87.0%) | 28 (22-31) | 40 (30-70) | 60 (24-96), n=21 |
| C | No | 66 (74.2%) | 44 (31-51) | 49 (74.2%) | 28 (23-31) | 40 (20-65) | 48 (21-164), n=64 |
|  | Yes | 23 (25.8%) | 47 (37-53) | 16 (72.7%) | 29 (26-30) | 38 (25-60) | 48 (24-54), n=21 |
| D | No | 17 (38.6%) | 40 (33-44) | 16 (94.1%) | 26 (22-30) | 35 (25-50) | 48 (24-48), n=9 |
|  | Yes | 27 (61.4%) | 43 (34-54) | 23 (85.2%) | 25 (22-31) | 45 (35-60) | 26 (15-48), n=19 |
| E | No | 51 (38.1%) | 42 (30-50) | 45 (88.2%) | 29 (24-32) | 40 (25-60) | 24 (15-60), n=36 |
|  | Yes | 83 (61.9%) | 43 (32-54) | 63 (75.9%) | 28 (27-31) | 39 (25-50) | 29 (9-60), n=46 |
| F | No | 20 (25.6%) | 38 (28-53) | 17 (85.0%) | 30 (23-33) | 48 (18-68) | 40 (12-78), n=20 |
|  | Yes | 58 (74.4%) | 40 (31-50) | 52 (89.7%) | 30 (27-32) | 35 (20-55) | 30 (12-84), n=57 |
| G | No | 24 (77.4%) | 42 (31-56) | 18 (75.0%) | 29 (25-31) | 43 (20-60) | 54 (10-120), n=12 |
|  | Yes | 7 (22.6%) | 37 (33-49) | 6 (85.7%) | 24 (24-28) | 50 (25-70) | 24 (18-48), n=5 |
| H | No | 74 (55.2%) | 36 (29-48) | 63 (85.1%) | 29 (26-32) | 40 (20-55) | 24 (12-48), n=67 |
|  | Yes | 60 (44.8%) | 43 (31-56)* | 47 (78.3%) | 28 (25-31) | 40 (30-60) | 21 (12-49), n=56 |
| I | No | 24 (48.0%) | 37 (28-47) | 18 (75.0%) | 27 (25-33) | 45 (27-58) | 24 (12-60), n=22 |
|  | Yes | 26 (52.0%) | 41 (34-49) | 23 (88.5%) | 27 (24-32) | 38 (20-60) | 42 (21-108), n=20 |
| J | No | 130 (71.8%) | 41 (30-49) | 94 (72.3%) | 28 (23-31) | 50 (25-70) | 60 (24-120), n=110 |
|  | Yes | 51 (28.2%) | 45 (31-50) | 42 (84.0%) | 27 (24-30) | 50 (30-65) | 25 (17-108), n=47 |
| K | No | 11 (35.5%) | 30 (24-35) | 10 (90.9%) | 30 (25-33) | 35 (15-45) | 60 (13-120), n=11 |
|  | Yes | 20 (64.5%) | 40 (26-50) | 16 (80.0%) | 29 (25-31) | 35 (23-40) | 42 (18-96), n=18 |
| Overall | No | 512 (53.7%) | 39 (29-49) | 409 (79.9%) | 28 (24-31) | 45 (21-65) | 36 (18-84), n=418 |
|  | Yes | 442 (46.3%) | 42 (32-51)* | 360 (81.8%) | 28 (25-31) | 40 (25-60) | 26 (12-80), n=345* |

* Evidence of difference (p≤0.05) by Kruskal-Wallis test (medians) or Fisher’s exact test (proportions).

Table S2: Treatments received by newly-referred patients across CFS/ME specialist services (n/a = data not available)

| Service |  | Individual face-to-face sessions | Individual telephone or Skype sessions | Group sessions | Occupational therapist | Physiotherapist | Other† | CBT | GET |
| --- | --- | --- | --- | --- | --- | --- | --- | --- | --- |
| A | Treatment data available, N=126 | 46.0% (325/707) | 5.8% (41/707) | 48.2% (341/707) | n/a | n/a | n/a | n/a | n/a |
|  | With follow-up data (64/126 (50.8%)) | 45.8% (158/345) | 4.3% (15/345) | 49.9% (172/345) | n/a | n/a | n/a | n/a | n/a |
|  | Discharged before follow-up (41/64 (64.1%)) | 38.8% (59/152) | 0.0% | 61.2% (93/152) | n/a | n/a | n/a | n/a | n/a |
| B | Treatment data available, N=55 | 65.5% (255/389) | 0.0% | 34.4% (134/389) | 43.7% (150/343) | 29.2% (100/343) | 27.1% (93/343) | n/a | n/a |
|  | With follow-up data (23/55 (41.8%)) | 68.6% (129/188) | 0.0% | 31.4% (59/188) | 35.4% (57/161) | 34.2% (55/161) | 30.4% (49/161) | n/a | n/a |
|  | Discharged before follow-up (5/23 (21.7%)) | 100% (25/25) | 0.0% | 0.0% | 42.3% (11/26) | 57.7% (15/26) | 0.0% | n/a | n/a |
| C | Treatment data available, N=22^#^ | 15.5% (34/200) | 0.0% | 84.5% (186/220) | 43.6% (96/220) | 10.0% (22/220) | 46.4% (102/220) | n/a | n/a |
|  | Discharged before follow-up (7/22 (31.8%)) | 15.9% (10/63) | 0.0% | 84.1% (53/63) | 15.9% (10/63) | 12.7% (8/63) | 71.4% (45/63) | n/a | n/a |
| D | Treatment data available, N=42 | 80.5% (177/220) | 19.5% (43/220) | 0.0% | n/a | n/a | n/a | 13.1% (23/176) | 86.9% (153/176) |
|  | With follow-up data (25/42 (59.5%)) | 82.6% (133/161) | 17.4% (28/161) | 0.0% | n/a | n/a | n/a | 15.7% (21/134) | 84.3% (113/134) |
|  | Discharged before follow-up (13/25 (52.0%)) | 72.1% (44/61) | 27.9% (17/61) | 0.0% | n/a | n/a | n/a | 22.6% (12/53) | 77.4% (41/53) |
| E | Treatment data available, N=133 | 88.7% (662/746) | 0.0% | 11.3% (84/746) | 77.3% (577/746) | 0.8% (6/746) | 21.8% (163/746) | n/a | n/a |
|  | With follow-up data (83/133 (62.4%)) | 92.4% (453/490) | 0.0% | 7.6% (37/490) | 73.7% (361/490) | 0.8% (4/490) | 25.5% (125/490) | n/a | n/a |
|  | Discharged before follow-up (26/83 (31.3%)) | 84.8% (128/151) | 0.0% | 15.2% (23/151) | 82.1% (124/151) | 1.3% (2/151) | 16.6% (25/151) | n/a | n/a |
| F | Treatment data available, N=78 | 58.6% (534/912) | 5.2% (47/912) | 36.3% (331/912) | n/a | n/a | n/a | n/a | n/a |
|  | With follow-up data (58/78 (74.4%)) | 60.6% (448/739) | 2.4% (18/739) | 36.9% (273/739) | n/a | n/a | n/a | n/a | n/a |
|  | Discharged before follow-up (27/58 (46.6%)) | 51.1% (142/278) | 0.0% | 48.9% (136/278) | n/a | n/a | n/a | n/a | n/a |
| G | Treatment data available, N=25 | 97.9% (319/330) | 0.0% | 2.1% (7/326) | n/a | n/a | n/a | n/a | n/a |
|  | With follow-up data (7/25 (28.0%)) | 94.1% (95/101) | 0.0% | 5.9% (6/101) | n/a | n/a | n/a | n/a | n/a |
|  | Discharged before follow-up (4/7 (57.1%)) | 88.2% (45/51) | 0.0% | 11.8% (6/51) | n/a | n/a | n/a | n/a | n/a |
| H | Treatment data available, N=133 | n/a | n/a | n/a | 55.4% (419/756) | - | 1.3% (10/756) | 43.3% (327/756) | n/a |
|  | With follow-up data (60/133 (45.1%)) | n/a | n/a | n/a | 58.6% (241/411) | - | 0.7% (3/411) | 40.6% (167/411) | n/a |
|  | Discharged before follow-up (19/60 (31.7%)) | n/a | n/a | n/a | 65.4% (87/133) | - | 33.8% (45/133) | 0.8% (1/133) | n/a |
| I | Treatment data available, N=50 | 22.4% (70/313) | 0.0% | 77.6% (243/313) | 10.0% (31/310) | 5.2% (16/310) | 84.8% (263/310) | n/a | n/a |
|  | With follow-up data (26/50 (52.0%)) | 18.9% (39/206) | 0.0% | 81.1% (167/206) | 11.3% (23/204) | 3.9% (8/204) | 84.8% (173/204) | n/a | n/a |
|  | Discharged before follow-up (4/26 (15.4%)) | 100% (16/16) | 0.0% | 0.0% | 43.8% (7/16) | 31.3% (5/16) | 25.0% (4/16) | n/a | n/a |
| J | Treatment data available, N=47^#^ | 53.2% (159/299) | 0.0% | 46.8% (140/299) | 5.7% (16/280) | 0.0% | 94.3% (264/280) | n/a | n/a |
|  | Discharged before follow-up (23/47 (31.8%)) | 73.0% (54/74) | 0.0% | 27.0% (20/74) | 0.0% | 0.0% | 100.0% (74/74) | n/a | n/a |
| K | Treatment data available, N=31 | 42.6% (156/366) | 0.0% | 57.4% (210/366) | 79.5% (291/366) | 0.0% | 20.5% (75/366) | n/a | n/a |
|  | With follow-up data (20/31 (64.5%)) | 46.1% (119/258) | 0.0% | 53.9% (139/258) | 82.6% (213/258) | 0.0% | 17.4% (45/258) | n/a | n/a |
|  | Discharged before follow-up (9/20 (45.0%)) | 35.1% (33/94) | 0.0% | 64.9% (61/94) | 67.0% (63/94) | 0.0% | 33.0% (31/94) | n/a | n/a |

† Other: Service B, joint occupational therapist (OT) + physiotherapist (PT) sessions; Service C, Rehabilitation Assistant; Service E, nurse, clinician or dietician; Service I, mostly joint OT/PT session (66/310), psychologist only (46/310), joint OT/psychologist (105/310) or joint PT/psychologist (23/310); Service J, mostly multidisciplinary team (186/280), psychologist ((22/280) and nurse (44/280); Service K, mostly joint OT/PT (39/366) or psychologist (24/366).

# Treatment data were only extracted for patients who had 1-year follow-up data

‡ Including 67 domiciliary visits

Table S3: Mean change (95% CI) in patient-reported outcome measures between assessment and 1-year follow-up, categorised by overall improvement in health

|  | Very much better (n=25) | Much better (n=92) | A little better (n=157) | No change (n=65) | A little worse (n=51) | Much worse (n=23) | Very much worse (n=11) |
| --- | --- | --- | --- | --- | --- | --- | --- |
| Chalder Fatigue Scale (range 0-33) | -14.9  (-18.1, -11.7) | -11.0  (-12.5, -9.50) | -5.90  (-6.97, -4.82) | -2.28  (-3.59, -0.96) | -1.24  (-3.08, 0.61) | -0.26  (-2.81, 2.29) | -2.27  (-4.77, 0.22) |
| SF36 Physical Function Subscale (range 0-100) | 29.8 (21.4, 38.2) | 17.4 (13.2, 21.6) | 0.96 (-2.14, 4.06) | 1.29 (-3.80, 6.39) | -7.67 (-12.0, -3.30) | -9.35 (-16.5, -2.21) | -15.0 (-25.5, -4.51) |
| Work & Social Adjustment Scale (range 0-40) | -13.8 (-17.6, -10.1) | -8.64 (-10.2, -7.12) | -2.12 (-3.20, -1.05) | -1.89 (-3.30, -0.48) | 0.38 (-1.45, 2.21) | 3.27 (0.88, 5.67) | 2.55 (-0.60, 5.69) |
| Visual analogue pain rating scale (range 0-100) | -30.9 (-41.9, -19.9) | -14.4 (-20.1, -8.78) | -1.58 (-5.62, 2.46) | -1.67 (-7.46, 4.11) | 5.65 (-0.24, 11.5) | 8.58 (0.69, 16.5) | 9.56  (-1.27, 20.4) |
| HADS Anxiety Score (range 0-21) | -4.32 (-6.35, -2.29) | -1.98 (-2.82, -1.13) | -0.24 (-0.80, 0.32) | 0.23 (-0.60, 1.06) | 0.49 (-0.36, 1.34) | -0.35 (-2.11, 1.41) | 2.3  (-0.16, 4.76) |
| HADS Depression Score (range 0-21) | -5.83 (-7.64, -4.02) | -3.25 (-3.92, -2.57) | -0.91 (-1.41, -0.42) | -0.94 (-1.66, -0.21) | 0.82 (-0.13, 1.78) | 1.10 (-0.15, 2.35) | 0.90  (-1.69, 3.49) |
| Epworth Sleepiness Scale (range 0-24) | -3.24 (-5.17, -1.31) | -2.50 (-3.48, -1.52) | -0.59 (-1.32, 0.13) | 0.10 (-0.81, 1.01) | -0.47 (-1.53, 0.60) | -1.00 (-2.45, 0.45) | 1.3  (-1.71, 4.31) |
| Jenkins Sleep Scale (range 0-20) | -3.59 (-6.09, -1.09) | -3.61 (-4.46, -2.75) | -1.04 (-1.81, -0.28) | -0.70 (-1.80, 0.39) | 0.69 (-0.16, 1.54) | 1.20 (-0.65, 3.05) | -1.10  (-4.25, 2.05) |
| CIS20R Fatigue Subscale (range 8-56) | -18.8 (-23.2, -14.3) | -10.8 (-12.7, -8.90) | -3.59 (-4.71, -2.48) | -0.02 (-1.30, 1.27) | 0.65 (-0.48, 1.79) | 0.40 (-1.51, 2.31) | 1.50  (-2.55, 5.55) |
| CIS20R Concentration Subscale (range 5-35) | -8.77 (-12.8, -4.75) | -6.41 (-7.84, -4.97) | -1.98 (-2.95, -1.01) | 0.23 (-1.10, 1.55) | 0.07 (-1.25, 1.38) | -0.55 (-2.49, 1.39) | -0.30  (-2.76, 2.16) |
| CIS20R Motivation Subscale (range 4-28) | -7.45  (-10.1, -4.84) | -5.19 (-6.42, -3.95) | -1.76 (-2.58, -0.95) | -1.00 (-2.07, 0.07) | 0.04 (-1.28, 1.36) | 0.05 (-1.48, 1.58) | 0.10  (-3.08, 3.28) |
| CIS20R Activity Subscale (range 3-21) | -6.59 (-9.01, -4.17) | -5.01 (-6.26, -3.76) | -1.54 (-2.32, -0.75) | 0.00 (-1.13, 1.13) | 0.04 (-1.08, 1.16) | 0.42 (-1.97, 2.81) | 3.20  (-0.24, 6.64) |

Table S4: Baseline characteristics of former patients with and without 2- to 5-year follow-up data across CFS/ME specialist services (N=1265)

| Service | 2- to 5-year follow-up | Number of patients | Age (years) | Female | Chalder Fatigue score (range 0-33) | SF-36 physical function score (range 0-100) | Duration of illness (months) |
| --- | --- | --- | --- | --- | --- | --- | --- |
|  |  | n (%) | median (IQR) | n (%) | median (IQR) | median (IQR) | median (IQR) |
| A | No | 154 (77.8%) | 40 (30-49) | 117 (76.0%) | 27 (25-30) | 47 (27-67) | 24 (10-60) |
|  | Yes | 44 (22.2%) | 47 (35-52)* | 35 (76.0%) | 29 (25-31) | 45 (27-60) | 26 (12-132) |
| B | No | 102 (61.8%) | 38 (32-45) | 77 (75.5%) | 28 (26-31) | 52 (22-67) | 36 (15-84) |
|  | Yes | 63 (38.2%) | 42 (35-50)* | 53 (84.1%) | 27 (23-30) | 52 (37-72) | 24 (14-60) |
| E | No | 159 (67.4%) | 39 (29-49) | 124 (78.0%) | 28 (24-31) | 42 (27-62) | 30 (12-96) |
|  | Yes | 77 (32.6%) | 45 (30-53) | 52 (67.5%) | 28 (23-32) | 32 (22-57) | 30 (12-84) |
| G | No | 136 (71.6%) | 36 (30-44) | 107 (78.7%) | 27 (24-31) | 47 (27-67) | 36 (18-120) |
|  | Yes | 54 (28.4%) | 39 (30-48) | 40 (74.1%) | 27 (23-30) | 52 (32-67) | 40 (10-96) |
| H | No | 149 (65.6%) | 41 (32-49) | 122 (81.9%) | 28 (25-31) | 42 (22-67) | 36 (12-90) |
|  | Yes | 78 (34.5%) | 43 (33-51) | 63 (80.8%) | 28 (23-31) | 47 (27-62) | 48 (20-108) |
| J | No | 142 (71.4%) | 36 (28-43) | 112 (78.9%) | 28 (24-31) | 47 (22-67) | 51 (19-102) |
|  | Yes | 57 (28.6%) | 44 (33-51)* | 40 (70.2%) | 27 (24-30) | 47 (37-62) | 49 (18-120) |
| K | No | 38 (76.0%) | 37 (30-43) | 31 (81.6%) | 27 (25-31) | 42 (27-67) | 26 (18-93) |
|  | Yes | 12 (24.0%) | 42 (40-50)* | 10 (83.3%) | 29 (25-30) | 30 (25-40) | 48 (27-120) |
| Overall | No | 880 (69.6%) | 38 (30-47) | 690 (78.4%) | 28 (24-31) | 47 (27-67) | 36 (14-96) |
|  | Yes | 385 (30.4%) | 43 (33-51)* | 293 (76.1%) | 28 (23-30) | 47 (27-62) | 36 (15-98) |

* Evidence of difference (p≤0.05) by Kruskal-Wallis test (medians) or Fisher’s exact test (proportions)
