# Supplementary figures and images for: Specialist treatment of chronic fatigue syndrome/ME: a cohort study among adult patients in England
Source: BMC Health Serv Res. 2017 Jul 14;17:488. doi: 10.1186/s12913-017-2437-3 (PMC5513420; doi:10.1186/s12913-017-2437-3)

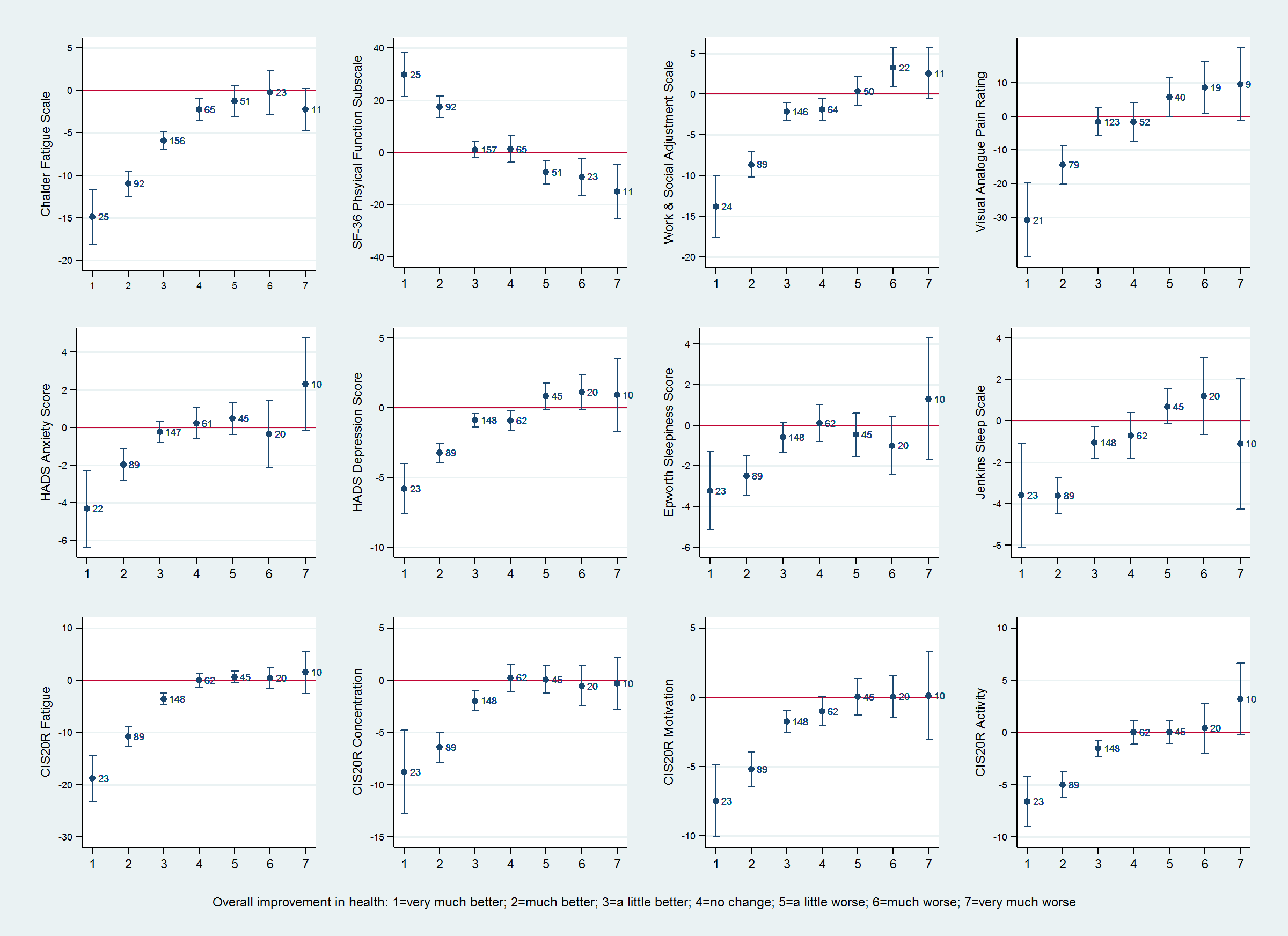

Supplement: Supplementary file 2 — Changes (mean difference with 95% CI) in patient reported measures between initial assessment and 1-year follow-up by overall improvement in health among patients treated by CFS/ME specialist services. (TIFF 12271 kb) [file 12913_2017_2437_MOESM2_ESM.tif]
